# Complications of Subcutaneous Contraception: A Review

**DOI:** 10.7759/cureus.2132

**Published:** 2018-01-31

**Authors:** Rebecca C Ramdhan, Emily Simonds, Charlotte Wilson, Marios Loukas, Rod J Oskouian, R. Shane Tubbs

**Affiliations:** 1 Department of Anatomical Sciences, St. George's University School of Medicine, Grenada, West Indies; 2 Seattle Science Foundation; 3 Neurosurgery, Swedish Neuroscience Institute; 4 Neurosurgery, Seattle Science Foundation

**Keywords:** subdermal, contraceptives, subcutaneous, neuropathy

## Abstract

Over 62 million women in the United States are of childbearing age and 60% of them use contraception. Subcutaneous contraceptives include implantable contraceptives and subcutaneous injections. Implantable contraception involves subdermal time-release of synthetic progestin, which allows for several years of continuous, highly effective contraception. Its main effects are inhibition of ovulation and thickening of the cervical mucus. Many complications have been associated with subcutaneous contraception, including menstrual disturbances, headache, weight gain, acne, dizziness, mood disturbances, nausea, lower abdominal pain, hair loss, loss of libido, pain at the implant site, neuropathy, and follicular cysts. Using standard search engines, the complications of subcutaneous contraception are reviewed. Patients should be adequately counseled on the possible complications and side effects of subcutaneous contraception to help them make an informed decision when choosing the right contraceptive to meet their needs.

## Introduction and background

Contraception is defined as any method that prevents conception or childbirth. Numerous medical options and devices can be used for this purpose. They can be classified as reversible (behavioral, barrier, and hormonal) and permanent methods. Hormonal methods include oral contraceptive pills, transdermal patches, vaginal rings, intrauterine devices, emergency contraception, injectable contraception, and implantable contraceptives. This review focuses on the medical complications of subcutaneous contraception, including injectable and implantable contraceptives/subdermal methods.

## Review

Depot medroxyprogesterone acetate

Depot medroxyprogesterone acetate (DMPA), also called Depo-Provera, can be administered intramuscularly (DMPA-IM) or via a subcutaneous injection (DMPA-SC). Medroxyprogesterone acetate (MPA) is a derivative of progesterone, the active agent in this form of contraception. The subcutaneous formulation contains 104 mg of the active drug, MPA, whereas the intramuscular formulation contains 150 mg of MPA [1]. Like other forms of progestin-only contraception, DMPA suppresses gonadotropin surges (luteinizing hormone and follicular stimulating hormone), preventing follicular maturation. This also creates a physical barrier that prevents sperm from entering the upper reproductive tract by increasing the viscosity of the cervical mucus. The progestin-only contraceptive methods slow tubal and endometrial mobility and make the lining of the endometrium much thinner than normal [2-3].

DMPA-SC effectively suppresses ovulation for at least 13 weeks in women. A report of two multinational and multicenter contraceptive efficacy trials demonstrated no pregnancies amongst 16,023 woman-cycles of exposure. These women represented a wide geographical region (Asia, Europe, and North and South America) and a broad range of weights (77-364 lbs) [4]. In contrast, DMPA-IM has a perfect-use failure rate of 0.3% and a typical-use failure rate of 3% in the first year [5].

Subdermal implants

Subdermal contraceptive implants have become popular contraceptive methods since they were introduced in the United States in 1991. They are safe and effective in preventing pregnancy [6]. The implant contraception provides sustained slow release of the steroid progestin, which results in anovulation, thinning of the endometrial lining, and thickening of the cervical mucus, creating a barrier that is impenetrable to sperm [7].

Levonorgestrel-releasing Implants: There are two types of levonorgestrel (LNG)-releasing implants, Norplant (Wyeth Ayerst Madison, NJ, USA) and Jadelle (Bayer, Turku, Finland). Both are inserted subdermally under local anesthesia, usually in the inner region of the nondominant arm. They release LNG continuously after insertion. Norplant is made up of six silastic capsules and Jadelle consists of two silastic rods. The release rates for Norplant are 85, 50, and 30 µg/day during the first, ninth, and 16^th^ month of usage, respectively. At the 16th month, 69% of the original steroid still remains in the capsules, which provides a safety margin for patients who do not return on time to replace their contraceptive. The insertion and removal of Jadelle are simpler because it has fewer units but achieves the same performance as Norplant. Sivin et al. (1997) investigated both modalities over a three-year period and found that the serum LNG levels in both systems were almost identical [8]. Levonorgestrel is very effective because of its strong antiestrogenic properties. It inhibits ovulation, prevents normal sperm transport through the female genital tract, and causes the inadequate development of the secretory endometrium [9].

Implanon (Merck Whitehouse Station, NJ, USA): Implanon is a single implant with an easy insertion and removal mechanism. The average serum level of etonogestrel with Implanon is 450 pg/ml, which decreases steadily to about 200 pg/ml at the end of three years [9]. It effectively inhibits ovulation in nearly 100% of cycles in all women. Not a single pregnancy has been reported in over 5,000 woman-years of exposure [10].

Nestorone (Zhejiang Xianju Pharmaceutical Co., Ltd. Xianju, Zhejiang, China): Nestorone is a progesterone derivative, 16-methylene-17-alpha-acetoxy-19-norprogesterone. Nestorone is inactive via the oral route, but with subcutaneous administration, it exerts a stronger progestational activity than LNG or desogestrel and can be used for contraceptive purposes. It lacks estrogenic, androgenic, and glucocorticoid activities [11].

According to Croxatto (2000), only one pregnancy was reported in roughly 4,000 woman-months of exposure, which occurred in one of the lower dosage groups. A single Nestorone implant that releases 150 µg/day can provide high contraceptive efficacy for two years with a six-month margin safety. Because oral ingestion inactivates it, Nestorone allows for satisfactory fertility regulation in breastfeeding mothers, as a transfer to the baby through breast milk will have no effect [10].

Uniplant: Uniplant, a progestin implant that releases nomegestrol acetate, was designed to be effective for one year [11]. Benagiano et al. (2008) reported that the contraceptive effect of Uniplant involves at least three different endocrine mechanisms: an inadequate luteal phase, prevention of follicular growth, and development of a persistent non-luteinized follicle [11].

Medical complications

The medical complications associated with contraceptive implants include menstrual disturbances, headache, weight gain, acne, dizziness, mood disturbances, nausea, lower abdominal pain, hair loss, loss of libido, pain at the implant site, neuropathy, and follicular cysts [12].

Menstrual Disturbances: In animal studies, normal menstruation occurs mainly from spiral arterioles and is initially controlled by vasoconstriction [13]. In contrast, breakthrough bleeding arises from small vessels on the endometrial surfaces, which consist of endothelial cells surrounded by a basement membrane and pericytes. Evidence demonstrates that the integrity of these small vessels is compromised by contraceptive implants [14]. During the initial exposure to Norplant, the supporting structures around the vessels are reduced, making the vessels more fragile, which explains the tendency of breakthrough bleeding during the first few months. The progesterone-exposed endometrium has less vascular support from the pericytes because the vascular smooth muscle is reduced [15]. Norplant also interacts with cytokeratin in the endometrial epithelium, making the blood vessels more fragile, causing them to leak into the endometrial cavity [16]. 

Several studies have investigated the relationship between bleeding patterns and ovarian function, endometrial thickness and serum LNG levels. Women can experience a variety of bleeding patterns despite similar hormonal levels. For example, low estradiol levels and an absence of luteal activity can be associated with amenorrhea, frequent or prolonged bleeding [17]. Those with regular bleeding patterns can have ultrasound images and estradiol and progesterone levels compatible with normal ovulation, luteinized unruptured follicles, persistent enlarged follies without ovulation, or even no follicular development [18-19].

In some studies, bleeding occurred after a rise and fall in serum estradiol or both estradiol and progesterone. Prolonged bleeding was often concomitant with a drop in estradiol levels, ending with a rise in estradiol levels [17]. In patients who had anovulatory cycles with Nestorone implants, the bleeding period was significantly associated with the highest concentration of estradiol during the previous 15 days [20].

Studies show that patients on Norplant have a thinner endometrium regardless of bleeding patterns or hormonal levels (estradiol and progesterone) [21]. There is a positive association between endometrial thickness, peripheral estradiol levels, and number of bleeding days during the 90 days before endometrial biopsy [22]. Darney et al. found that a proliferative endometrium was associated with abnormal bleeding in Norplant patients [23].

Vaginal bleeding pattern disturbances among Norplant users tend to be most common during the first few months of use and are likely to diminish over time owing to a decrease in the rate of LNG release, allowing ovulatory-like cycles to return [24]. According to Fraser et al., most women experience prolonged bleeding at irregular intervals during the initial year, while 25% have regular bleeding and approximately 10% have amenorrhea [25]. After the initial year, there are decreases in the number of bleeding episodes and days and in the length of bleeding periods; by the fifth year, 66% experience regular bleeding patterns, 33% experience irregular patterns, and amenorrhea is not common [26].

Implanon causes substantial vaginal bleeding irregularities, amenorrhea occurs in 30%-40% of patients during the initial three months and subsequently remains around the same level. Half of the users experience infrequent bleeding during the first three months but this decreases to 30% by the sixth month. Approximately 30% of patients had prolonged bleeding, which later declined to less than 20% [14]. Implanon has been shown to cause more amenorrhea and less frequent bleeding than Norplant does, whereas the incidence of prolonged bleeding is similar in both methods and decreases with time [27].

Headache: According to Brache et al., headache seems to be the most common complaint among patients using progestin implants [12]. There is wide variability among studies reporting complaints of headaches, ranging between three percent and 69% of patients. The higher scores were reported by Cullins et al.: 43%, Frank et al. : 49%, and Croxatto et al.: 69%. [28-30]. Headaches account for approximately 10%-20% of all medical reasons for the removal and discontinuation of progestin implants, excluding menstrual disturbances. Less than five percent of all users discontinue progestin implants because of headaches. The exact etiology is unknown but is probably steroid-related [12].

Weight Changes: Weight gain is also a very common complaint among users. Every long-term study focusing on any contraceptive method demonstrates an increase in the average weight of users, but it is difficult to determine how much of that weight gain is related to the method of contraception. Most studies exploring body weight have shown an average increase of 0.4-1.5 kg/year with all implant systems [12]. A controlled study by Sivin et al. reported a 0.7 kg/year weight gain in both Norplant and intrauterine device (IUD) users, while another controlled study found a 2.5 kg weight gain over five years in Chinese women [24]. However, the latter was only 1.0 kg higher than the increase in nonhormonal controls according to the International Collaborative Post-Marketing Surveillance of Norplant, 2001. Weight gain resulted in the removal of implants in approximately 0.5%-5.6% of patients [12].

Weight loss was reported by 3.5% of users and was responsible for discontinuation rates of 0.2%-1.2% [12]. A small study noted a 0.8 kg weight loss after one year of Norplant use compared to a 0.06 kg increase among DMPA users [31].

Acne, Hair Loss, and Hirsutism: Generally, acne, hair loss, and hirsutism are attributed to the androgenic effects of progestogens and were found to be more common among Norplant implants users than nonhormonal controls [12].

Acne has been reported in approximately 3%-27% of patients. More than half of the women with pre-existing acne reported an improvement during usage, while the condition worsened in about 10% [32].

Hair loss and hirsutism are not as common as acne. In two interview studies, 18%-19% of users reported hair loss or abnormal hair growth during the first year of use. In a study comparing Norplant with IUD users, patients more frequently experienced skin conditions (acne, hair loss, and hirsutism) with Norplant (8.1% versus 3.2%) during the first year. However, in the second year, this was reversed (7.9% versus 9.8%) [33]. The discontinuation rate due to acne ranged from 0%-2% whereas the discontinuation rate due to hair loss and hirsutism ranged from 0%-1% [12].

Dizziness: Reports indicate that 4%-11% of contraceptive implant users experience dizziness. The rate of dizziness tends to be higher in Norplant users than nonhormonal controls [34]. According to Brache et al., the annual occurrence rate was higher during the first year than subsequent years of use (5%-8% versus 3%-4%) [12]. According to Coutinho, dizziness rarely led to implant removal (0%-2.3%) [35].

Lower Abdominal Pain: Lower abdominal pain was apparent in 7%-23% percent of patients using Norplant, Jadelle, and Implanon, with an annual occurrence rate of 7.3%-9.6% for Norplant and Jadelle users. Less than 1.7% of women had their implants removed for this indication [8,10].

Ovarian Cysts: Ovarian function is not fully suppressed by exposure to continuous low-dose progestins, so gonadotropins are still released and dominant follicles develop. However, the normal ovulatory process is disrupted so the follicle does not rupture, as it normally would. The follicles become enlarged (>30 mm), and this gives the appearance of ovarian cysts, which should spontaneously disappear in one to two months [36-37].

A number of studies in which the ovaries were examined sonographically demonstrated persistent follicles in 56%-63% of cycles with the implant system, the maximum follicle diameter being less than 35 mm [12]. According to Alvarez-Sanchez et al., one study demonstrated that 17.6% of Norplant users but only 4% of IUD users had follicles with diameters greater than 25 mm [37].

A persistent follicle found in implant users on ultrasound examination warrants a one-month follow-up only. If it persists for more than two months, further studies are recommended, as it could indicate a serious pathology [12].

Mood Disturbances: Women using implantable contraception are more likely to report mood swings, nervousness, and depression than women using nonhormonal controls. With respect to removal rates, mood changes accounted for 0%-1.7% whereas nervousness led to 0.2%-1.6% and depression accounted for 0.2%-1.9% [12].

Nausea, Breast Tenderness, Loss of Libido, and Fatigue: Sivin reported that nausea affects approximately 4%-12% of different implant users [24], a multicenter comparative study showing 6.5% for Norplant and 3.8% for Implanon [32]. There was a higher annual rate during the first year (7.5 per 100 woman-years) than subsequent years (4.2 per 100 woman-years) in the US Norplant trial [38]. This differed from an international comparative study, which showed an annual rate of 2.6 per 100 woman-years for Norplant users versus 2.9 per 100 woman-years for Jadelle [8]. Removal rates for nausea were all less than 1.2% [12].

Breast tenderness has been reported in approximately 3%-16% percent of patients using progestogen implants, with removal rates accounting for less than 1%. Loss of libido was reported in approximately 2.0%-5.4% of implant users with removal rates between 0% and 0.8%. Fatigue has been reported in 2.5%-4.2% of Jadelle and Norplant users with removal rates less than 0.6% [12].

Local Reactions: Pain localized at the insertion site is a common complication of subdermal implants. It occurs in approximately 2% to 3% of all women and normally resolves by the third month [39].

Infection has also been reported, with an incidence ranging from 0% to 1.4% [12]. Most infections occur within the first two months after placement but some as late as two years after [39]. A complication that can arise from infection is implant expulsion, but spontaneous expulsion has also been reported in less than 0.6% of patients [12]. According to Klavon et al., 35.7% of expulsions took place within the first two months after placement and about 70% within the first four months [39].

According to Sivin et al., 10.6% of patients reported changes in the color of their skin over the implant site but this did not lead to discontinuation [40]. Another study reported that Jadelle led to tenderness, numbness, tingling, and hyperpigmentation in more than 5% of patients [40]. Alvarez et al. reported that 36% of their patients experienced hyperpigmentation and 20% of subjects experienced skin depression at the implant site [41]. The skin depression where the tubes are implanted has the appearance of loss of subcutaneous fat in that area. This complaint is more common among women with a larger body mass index (BMI), so it could be attributable to their thicker layer of subcutaneous fat. Hyperpigmentation appears to be related to the amount of melatonin in the skin. The exact etiology of these changes is unknown, but they could be related to the local release of the steroid or could be a direct result of a reaction to a foreign body [41].

Pregnancy: There have been over 200 reports of unintended pregnancies associated with Implanon, which occurred during the first three years' post-marketing in Australia [42]. Out of 218 cases, 84 were attributed to noninsertion (documented evidence that the implant had not been inserted, as it could not be located by palpation or ultrasound or there were negative serum etonogestrel levels). Nineteen unintended pregnancies were due to incorrect timing (Implanon was inserted outside the recommended times, i.e., not within the first five days of the menstrual cycle or 21-28 days postpartum). Drug interaction accounted for eight cases. Etonogestrel is normally metabolized by the CYP 3A4 enzyme, and in these eight cases, the patients were taking hepatic enzyme-inducing antiepileptic drugs, which lowered the serum etonogestrel levels. The Implanon was expelled in three cases, the product failed in 13 cases, and there was insufficient information in 45. Lastly, and interestingly, 46 cases of unintended pregnancies were due to prior conception, i.e. the dates of conception indicated that the patient was already pregnant at the time of insertion [42].

Peripheral Neuropathy: Peripheral neuropathy is a rare complaint among women using subdermal implants [28]. A case report by Hueston et al. discussed a 22-year-old woman who presented with left arm paresthesias 11 months after receiving a subdermal implant on that arm [43]. Internal rotation of her arms caused an electric shooting pain down the anterolateral aspect of her left arm to her fingers, which was associated with numbness and paresthesias. The pain lasted two to three minutes and could be reproduced by palpating the superior portion of the axillary subdermal capsule. Physical examination showed a subdermal implant extending from the mid-upper arm almost to the axilla, but there were no signs of inflammation (erythema, tenderness, or induration) over any of the capsule paths. Nevertheless, compression of the proximal portion of the most lateral subdermal capsule rekindled the symptoms [43].

In another case reported by Hueston et al., a 26-year-old woman presented with paresthesia and numbness that radiated down the lateral aspect of her arm to her left hand. She stated that one day previously, someone had grabbed her arm, causing immediate pain in the upper arm, and the next day, she experienced the paresthesia and numbness, which were transient and unrelated to arm movements. Compression of the site of trauma rekindled her symptoms. She had had a subdermal contraceptive implant placed one year earlier in that arm. Physical examination revealed no obvious sign of trauma but a subdermal implant was noted in the mid-upper arm radiating toward the axilla. Palpation of the implant produced no localized pain but palpation of the proximal tip of the capsule closest to the biceps muscle reproduced her symptoms. It was found that she had acute nerve compression from a capsule and edema from the soft tissue trauma. Her symptoms resolved with time [43].

Anatomically, the musculocutaneous nerve branches from the lateral cord of the brachial plexus just distal to the axilla. It then travels posterior to the coracobrachial muscle, penetrates the coracobrachialis, and extends to the anterior aspect of the arm. Symptoms are experienced along the anterolateral aspect of the lower arm to the hand if this nerve is compressed. The two case reports described above show a similar distribution of symptoms. In patients with short arms or in whom the subdermal capsule has been placed too proximally, the capsule’s tip can exert pressure on the nerve where it penetrates the coracobrachial muscle, producing these symptoms [43].

A case report by Marin et al. discussed a 27-year-old female who presented with numbness and tingling along the medial arm, forearm, and ulnar hand associated with decreased left-handed grip strength, and dexterity after her asymptomatic Norplant capsules had been removed because she desired pregnancy. A physical examination demonstrated clinical findings consistent with left ulnar nerve neuropathy and this was confirmed by nerve conduction studies, which revealed absent left ulnar motor and sensory conduction potentials across the Norplant removal site and scar. The patient underwent ulnar nerve epineurolysis, and a two-month follow-up demonstrated no improvement in the motor and sensory clinical findings, but she presented with the classic ‘claw hand’ indicative of advanced ulnar nerve palsy. After a six-month follow-up, there was only minimal, clinically insignificant, reinnervation of the ulnar hand muscles [7].

Another case report of a 23-year-old nulliparous demonstrated ulnar nerve injury associated with the removal of Norplant implants, which had been placed directly over the most anterior region of the triceps brachii muscle, the lower end of the implant being approximately five cm from the medial condyle of the elbow. The patient experienced a sharp, shooting pain down the medial lower arm to the ulnar-innervated digits (fourth and fifth digits) as soon as the needle was introduced. This pain was reproduced when another incision was made between the third and fourth implants, but no notable nerve injury was found during exploration. When the procedure was complete, the patient reported a mild, painful ‘tingly’ sensation but no weakness or extreme pain. Four days after implant removal, she had persistent pain and numbness over the hypothenar eminence along with the fourth and fifth digits and marked weakness of the ulnar-innervated muscles on the right hand. Further tests led to a definite diagnosis of an ulnar nerve injury [44]. After traumatic nerve injuries, motor function is initially reduced for three to five days whereas sensory function is reduced for five to eight days. Improved motor response at six weeks is a good prognostic sign for further recovery of ulnar nerve function [45].

Complications with implant insertions are rare, with an incidence of 0.3% [46]. Despite anatomical variations, a general rule is that the neurovascular structures should be avoided around the area of insertion, which is located below the deep fascia. The medial antebrachial nerve (medial cutaneous nerve of the forearm) arises from the medial cord of the brachial plexus, beginning between the axillary artery and the vein in the axilla, and provides sensory innervation to the anteromedial region of the forearm. It descends medial to the brachial artery and is closely associated with the basilic vein. The ulnar nerve descends posterior to the region between the biceps and triceps, beneath the deep fascia. The medial cutaneous nerve emerges through the deep fascia between the middle and lower arm and splits into anterior and posterior branches to cross the elbow. Insertion of the implant into the subcutaneous tissue about eight to 10 cm proximal to the medial epicondyle can help avoid injury to important neurovascular structures [47].

According to Sivin et al., insertion site complications are a result of poor surgical technique rather than Norplant capsules; 4.5%-7.5% of terminations of subdermal contraceptives are due to complications that arise from implant placement [48]. Insertion errors include placing the capsules either too superficially or too deeply. Placing the capsule in the dermis can lead to skin blistering [49]. If capsules are placed too deeply or too far apart, multiple removal attempts may be needed, which increases the risk of injury or infection [33].

## Conclusions

It is important for physicians and patients to be educated about the side effects that can arise from the use of subcutaneous contraceptives. Additionally, it is important to weigh the risks and benefits of each method. Physicians should be aware of the complications that can arise when inserting subcutaneous contraceptive devices, though rare complications can occur. Physicians should be familiar with the correct technique, including appropriate anatomical placement and depth.
